# Dynamic changes in heparin-binding protein as a prognostic biomarker for 30-day mortality in sepsis patients in the intensive care unit

**DOI:** 10.1038/s41598-022-14827-1

**Published:** 2022-06-24

**Authors:** Qing-Li Dou, Jiangping Liu, Wenwu Zhang, Ching-Wei Wang, Yanan Gu, Na Li, Rui Hu, Wan-Ting Hsu, Amy Huaishiuan Huang, Hoi Sin Tong, Tzu-Chun Hsu, Cheng-An Hsu, Jun Xu, Chien-Chang Lee

**Affiliations:** 1 Department of Emergency Medicine, The People’s Hospital of Baoan Shenzhen, Shenzhen, China; 2 Department of Emergency Medicine, The Second Affiliated Hospital of Shenzhen University, Shenzhen, China; 3 Health Data Science Research Group, Department of Emergency Medicine, The Centre for Intelligent Healthcare, National Taiwan University Hospital, No. 7, Chung-Shan South Road, Taipei, 100 Taiwan; 4 Department of Epidemiology, Harvard T.H. Chan School of Public Health, Boston, MA USA; 5 Li Ka Shing Faculty of Medicine, The University of Hong Kong, Hong Kong, China; 6 Department of Laboratory Medicine, National Taiwan University Hospital, Taipei, Taiwan; 7 Department of Emergency Medicine, Peking Union Medical College Hospital, Beijing, 100730 China

**Keywords:** Outcomes research, Predictive markers, Prognostic markers, Biomarkers, Infectious diseases, Bacterial infection

## Abstract

Heparin-binding protein (HBP) has been shown to be a robust predictor of the progression to organ dysfunction from sepsis, and we hypothesized that dynamic changes in HBP may reflect the severity of sepsis. We therefore aim to investigate the predictive value of baseline HBP, 24-h, and 48-h HBP change for prediction of 30-day mortality in adult patients with sepsis. This is a prospective observational study in an intensive care unit of a tertiary center. Patients aged 20 years or older who met SEPSIS-3 criteria were prospectively enrolled from August 2019 to January 2020. Plasma levels of HBP were measured at admission, 24 h, and 48 h and dynamic changes in HBP were calculated. The Primary endpoint was 30-day mortality. We tested whether the biomarkers could enhance the predictive accuracy of a multivariable predictive model. A total of 206 patients were included in the final analysis. 48-h HBP change (HBPc-48 h) had greater predictive accuracy of area under the curve (AUC: 0.82), followed by baseline HBP (0.79), PCT (0.72), lactate (0.71), and CRP (0.65), and HBPc-24 h (0.62). Incorporation of HBPc-48 h into a clinical prediction model significantly improved the AUC from 0.85 to 0.93. HBPc-48 h may assist clinicians with clinical outcome prediction in critically ill patients with sepsis and can improve the performance of a prediction model including age, SOFA score and Charlson comorbidity index.

## Introduction

Sepsis is a leading cause of morbidity and mortality, with in-hospital mortality ranging from 18 to 50%^1–3,4,5^. The incidence of sepsis has steadily increased in the past decade, from 143,000 admissions in 2000 to 343,000 in 2007 in the United States^6^. It is undeniably a major public health burden. The new SEPSIS-3 consensus defines sepsis as a “life-threatening organ dysfunction caused by a dysregulated host response to infection” and defines Septic shock as a “subset of sepsis in which particularly profound circulatory, cellular, and metabolic abnormalities are associated with a greater risk of mortality than with sepsis alone”^7^. Although the SOFA score had been used to predict outcome in septic patients, a recent prospective observational cohort study of 130 patients in Brazil suggests that a change in SOFA score at 48 h is only 61.3% sensitive in predicting the 30-day mortality^8^.

Heparin-binding protein (HBP), also called azurocidin or cationic antimicrobial protein, is a 37 kDa multifunctional protein contained within the secretory and azurophilic granules of polymorphonuclear leukocytes^9^. HBP worsens already dysregulated inflammatory responses and induces capillary leakage in septic patients^10,11^. There is exocytosis of 89% of the total HBP after only 30 min of phagocytosis of bacteria^11^. The rapid release of HBP can be explained by its location within the secretory granule, which is mobilized upon neutrophil activation. After release, HBP increases endothelial permeability^9–13^. Recently, HBP has been shown to be a robust predictor of the progression to organ dysfunction due to infection and sepsis^12–16^. HBP also rises earlier than organ dysfunction developed and increased plasma HBP was observed in over 90% of the patients who developed severe sepsis^17^.

Previous studies of other useful biomarkers in sepsis such as procalcitonin, have shown robust results that dynamic changes of the biomarker was a better predictor in survival than the absolute value at a given time point^18^. Therefore, in this study, we hypothesized that dynamic changes in HBP during sepsis may reflect both the severity of initial presentation and the response to the initial treatment^18^. We measure relative changes in HBP compared to the baseline values (HBP change, HBPc) and may predict outcome. This study aims to determine if dynamic changes in HBP can enhance the prediction of mortality in patients with sepsis. In this study, we prospectively evaluated the predictive value of day 1 (baseline), 24-h, and 48-h HBP changes for sepsis mortality. Moreover, we analyzed whether 48-h changes in HBP can enhance the predictive accuracy of a clinical prediction model.

## Methods

### Patient population and design

This study was approved by the Research Committees and Institutional Review Boards for People’s Hospital of Baoan District of ShenZhen, and written informed consents were obtained from patients or patient representatives. This study was performed in accordance with the principles of the Declaration of Helsinki and the Good Clinical Practice Guidelines. We conducted a prospective observational study in the Emergency Department Intensive Care Unit (EICU) of People’s Hospital of Baoan District of ShenZhen. Patients admitted to the EICU between August 2019 and January 2020 who fulfilled SEPSIS-3 criteria were enrolled^7^. Exclusion criteria included pregnancy, do-not-resuscitate orders, age under 20 years, leukopenia or hyperleukocytosis, hemolysis, and had received albumin or heparin treatment before HBP measurement. All septic patients were treated based on guidelines from the current Surviving Sepsis Campaign with modifications as deemed appropriate by the treating physicians^17–21^. The duration of antimicrobial therapy was guided by culture data, site of infection, and the treating physician. Patient data collected included: age, gender, vital signs, comorbidities, Acute Physiology and Chronic Health Evaluation (APACHE II) score, Sequential Organ Failure Assessment (SOFA) score, site(s) of infection, laboratory tests findings (basic biochemistry, complete blood count, coagulation, and arterial blood gas), microbiological culture results, duration of hospitalization (length of stay in ICU and in total), and clinical outcomes. Infection was diagnosed by clinical, laboratory, and microbiological parameters. APACHE II scores and SOFA scores were assessed on the first day of admission (day 1). Serum concentrations of procalcitonin (PCT) and lactate were measured on the first day of EICU admission. HBP was measured right after admission to EICU (baseline HBP) and was repeated at 24 and 48 h. Each patient had at least 3 HBP measurements, and were followed for 30 days, until death, discharge, or end of follow-up, whichever came first. The primary endpoint was all-cause mortality at 30 days.

### Blood sample collection and analysis

The blood samples were collected in 5 ml sodium citrate anticoagulation tubes (BD vacutainer) and centrifuged at 3000 rpm for 10 min immediately. The HBP level was determined by a commercial enzyme-linked immunosorbent assay kit (Joinstar Biomedical Technology Co., LTD, Hangzhou, China). The HBP detection range was 5.9–300 ng/mL. The intra-assay coefficient of variation was 11% at 21 ng/mL and 7% at 81 ng/mL. In addition, we investigated the prognostic ability of baseline PCT levels for mortality prediction. PCT level was measured via an automatic analyzer, the VIDAS® B.R.A.H.M.S PCT assay (bioMérieux, Marcy L'Etoile, France). The lower limit of detection of the assay was 0.01 ng/mL^22^.

### Plasma dynamic change of HBP

Change in plasma HBP was defined as the difference between a given timepoint and baseline (Day 1). In particular, we measured the 24-h plasma HBP change (HBPc-24 h) and the 48-h plasma HBP change (HBPc-48 h), both relative to the baseline (Day 1) measurement. The relative change of HBP was calculated by dividing the HBPc-24 h and HBPc-48 h by the baseline HBP, respectively, and was presented as a percentage. Baseline APACHE II score, Sequential Organ Failure Assessment (SOFA) score, primary source of infection, culture results, ICU, and hospital mortalities were recorded. Clinical parameters such as body temperature, heart rate, white cell count (WCC), and clinical signs of infection were recorded on admission and repeated daily^22^.

### Statistical analysis

To give an overview of the characteristics of the study population, we reported the demographic, comorbidity, sites of infection, clinical severity, and length of hospital stay for survivors and nonsurvivors. We then compared the characteristics between survivors and nonsurvivors. The categorical variables were expressed as absolute numbers and proportions and compared using Chi-square tests. Continuous variables were presented as median and interquartile range (IQR) and compared using the analysis of variance (ANOVA). For 21 patients who died before 48 h of admission, the missing HBP data at various time points were imputed using the machine learning algorithm (random forest). A Spearman's rank correlation test was used to assess the relationship between plasma concentrations of HBP and PCT. The discrimination of different biomarkers, or 24-h or 48-h HBP change (HBPc-24 h, HBPc-48 h) was assessed by area under the receiver operating characteristic (ROC) curves^23^. We first determined the best cutoff in terms of sensitivity and specificity by the Youden’s Index. Because cutpoints determined by the Youden index maximize the sum of sensitivity and specificity rather than individual sensitivity or specificity, we further determined the best cut points that maximized sensitivity/specificity with a given fixed value of sensitivity (0.9) or specificity (0.9)^24^. To assess whether the 48-h HBP change increases the predictive accuracy of empirical predictors. We built two Cox models for comparison. To assess whether the models meet the proportional hazards assumption, we used each predictor in our model as a time dependent covariate in Cox models. When the proportional assumption is violated, a time dependent covariate is significant in Wald test. The first model was an empirical model that includes three strong empirical predictors (age, Charlson score and SOFA score). These predictors were chosen based on previous studies^7,25^. The second model was a biomarker-enhanced one which added HBPc-48 h to the first model. The models were evaluated using c-statistics and calibration plots with Brier scores. A DeLong test was used to compare the c-statistics of the two models. Lastly, we created a nomogram of the biomarker-enhanced model. The nomogram could be used to calculate the estimated probability of mortality for a given patient. To further visualize the prognostic meaning of absolute value and clearance of HBP, we plotted Kaplan–Meier curves stratified by quartiles of the plasma levels of HBPc-48 h^26^. The survival difference of four quartile strata was compared using the log-rank test. For all statistical analyses, P < 0.05 was considered statistically significant. Data analysis and graphing were conducted with R statistical software (Foundation for Statistical Computing, Vienna, Austria).

### Ethics approval and consent to participate

This study was approved by the Research Committees and Institutional Review Boards for People’s Hospital of Baoan District of ShenZhen, and it met the criteria for exemption from informed consent.

This study was approved by the Research Committees and Institutional Review Boards for People’s Hospital of Baoan District of ShenZhen, and written informed consents were obtained from patients or patient representatives.

## Results

### Demographics and clinical presentations

**Figure 1:**
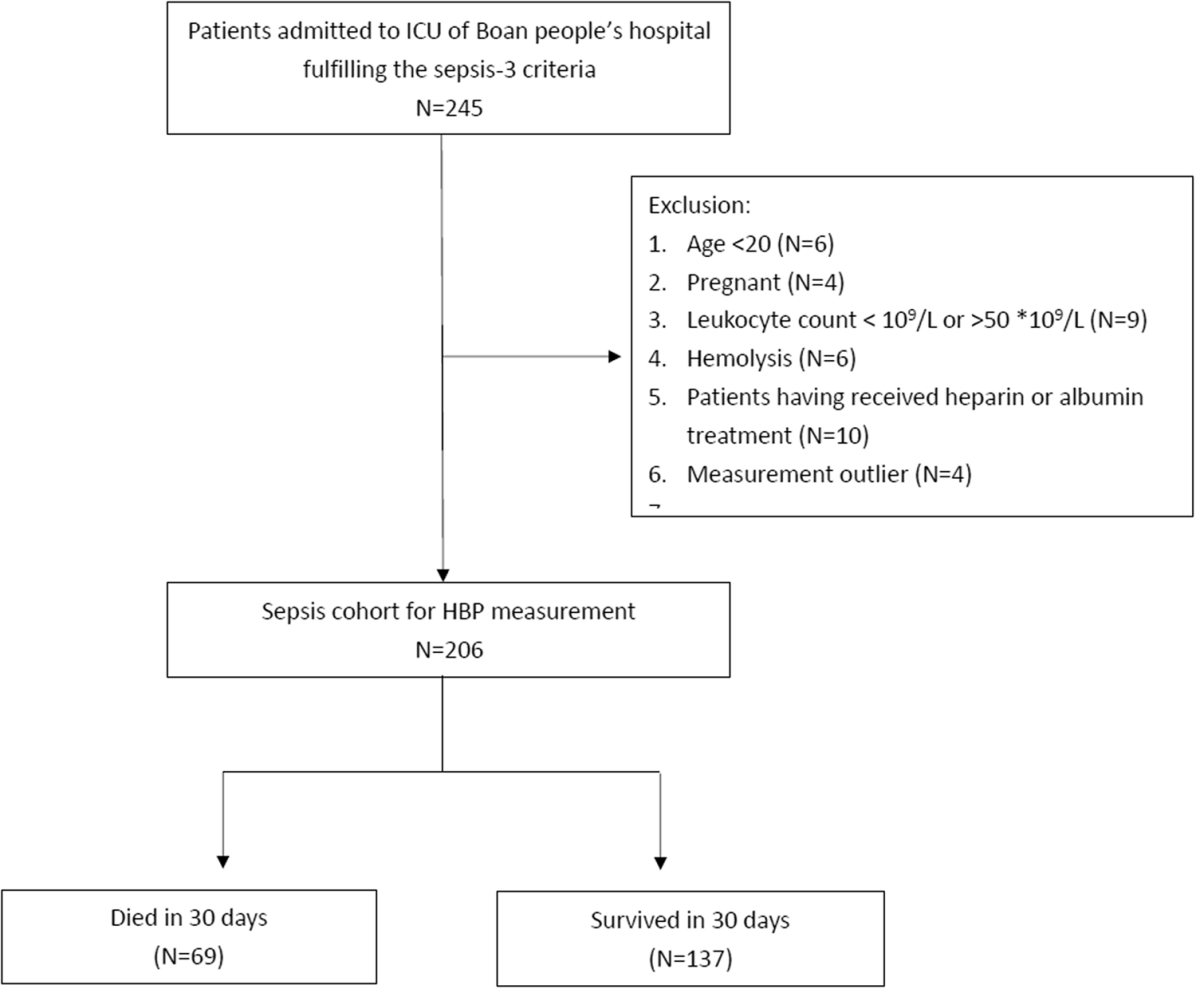
Cohort inclusion and exclusion process.

During the study period, a total of 245 patients were admitted for sepsis or septic shock. Six patients were excluded from the study due to age < 20 years, four due to pregnancy, nine due to leukopenia or hyperleukocytosis, six due to hemolysis, ten had received albumin or heparin treatment before HBP measurement, and four were measurement outliers. We included 206 patients including 21 patients who died within 48 h of admission for final analyses (Fig. 1).

To evaluate the baseline characteristic differences between survivors and nonsurvivors, we performed chi-square tests for categorical variables and analysis of variance (ANOVA) for continuous variables (Table 1). Compared to survivors, non-survivors were older, had higher serum levels of procalcitonin and lactate, and were more likely to have a pulmonary infection and positive blood cultures. Nonsurvivors also had higher APACHE II or SOFA scores and were more likely to require mechanical ventilation support. The comparison of patient characteristics across the two groups is summarized in Table 1.

**Table 1:** Comparisons of baseline characteristics and outcomes between survivors and nonsurvivors in patients with severe sepsis and septic shock.

	Survivors (N = 137)	Non-survivors (N = 69)	*p*-value
**Demographic characteristics**			
Age, median (IQR), year	65 (29)	61 (31)	0.607
Female, n (%)	48 (35)	22 (31.9)	0.768
**Comorbidities, n (%)**			
Chronic heart failure	13 (9.5)	19 (27.5)	0.002
Diabetes mellitus	31 (22.6)	15 (21.7)	1.000
Cerebrovascular disease	27 (19.7)	19 (27.5)	0.273
Chronic kidney disease	19 (13.9)	23 (33.3)	0.002
**Laboratory results, median (IQR)**			
White blood cell count (10^9^/L)	11.0 (7.06)	12.0 (9.49)	0.729
Neutrophil percentage (%)	86.0 (11.5)	87.7 (11.9)	0.504
Procalcitonin(ng/dL)	0.65 (5.86)	7.06 (27.70)	< 0.001
Lactate (mmol/L)	1.8 (1.7)	3.5 (5.1)	< 0.001
**Site of infection, n (%)**			
Bloodstream	2 (1.5)	9 (13.0)	0.002
Lung	112 (81.8)	68 (98.6)	0.001
Urinary tract	9 (6.6)	2 (2.9)	0.437
Abdomen	18 (13.1)	15 (21.7)	0.165
Soft tissue	2 (1.5)	1 (1.4)	1.000
Others	4 (2.9)	1 (1.4)	0.867
**Clinical scoring, points, median (IQR)**			
APACHE II score	14 (9)	25 (8)	< 0.001
SOFA score	5 (6)	13 (8)	< 0.001
Charlson score	2 (3)	4 (3)	< 0.001
Number dysfunctional organs	2(1)	4(2)	< 0.001
**Organ support, n (%)**			
Mechanical ventilation	21 (15.3)	30 (43.5)	< 0.001
Renal replacement therapy	6 ( 4.4)	28 (40.6)	< 0.001
Vasopressor	36 (26.3)	53 (76.8)	< 0.001
**Duration of hospitalization, mean ± SD, days**			
Length of ICU stay, median (IQR)	6 (8)	4 (12)	< 0.001
Length of hospital stay, median (IQR)	14 (14)	5 (12)	< 0.001

### Baseline and dynamic change of HBP among sepsis patients

**Figure 2:**
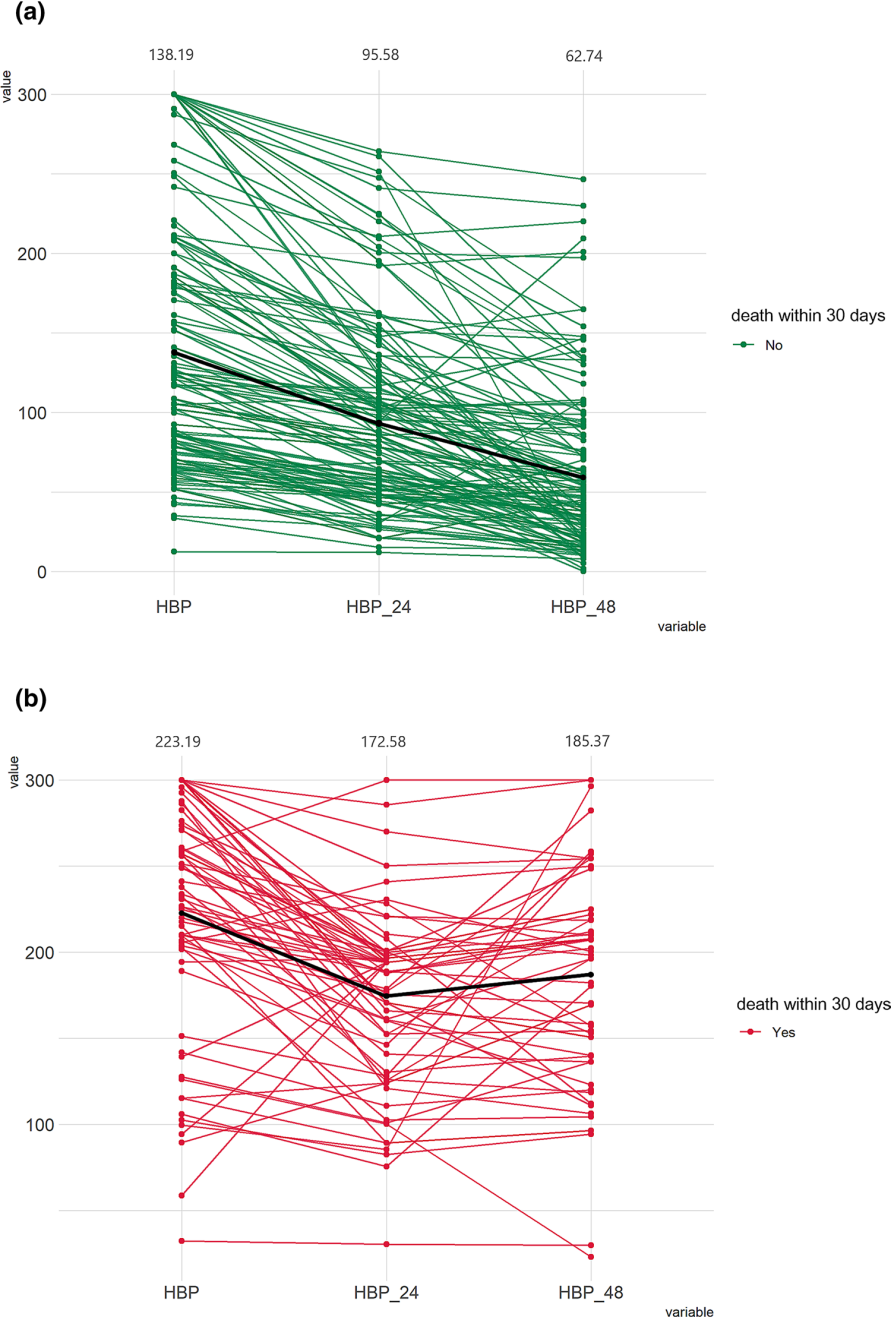
Serial measurement of plasma levels of HBP between sepsis survivors (**a**) and nonsurvivors (**b**).

Table 2 compares the baseline and dynamic changes of HBP within 48 h of admission between survivors and nonsurvivors by Mann–Whitney *U* tests. Median plasma levels of HBP were higher in nonsurvivors than survivors at admission (235 vs 117 (ng/mL), p < 0.001), 24 h (173 vs 85 (ng/mL), p < 0.001) and 48 h (196 vs 48 (ng/mL), p < 0.001). Survivors had significantly higher median 48-h HBP change (− 54 vs − 15(%), p < 0.001) than nonsurvivors. The time-dependent change of HBP between survivors and nonsurvivors is shown in Fig. 2 and Supplementary Fig. 1. Figure 2 illustrates the serial measurements of plasma level of HBP for each patient^22^. Nonsurvivors had higher admission HBP levels. At 24 h, both survivors and nonsurvivors had lower HBP levels. At 48 h, the decrease in HBP was more pronounced in survivors than nonsurvivors.

**Table 2:** Comparisons of HBP and HBPc dynamic monitoring levels between survivors and nonsurvivors in patients with sepsis or septic shock.

Variables	Survivors (N = 137)	Non-survivors (N = 69)	*p*-value
HBP-initial (ng/mL) median (IQR)	117 (75–185)	234 (203–276)	< 0.001
HBP-24 h (ng/mL) median (IQR)	85 (51–120)	188 (141–197)	< 0.001
HBP-48 h (ng/mL) median (IQR)	48 (24–86)	207 (151–210)	< 0.001
HBPc-24 h (%) median (IQR)	− 28 (− 42, − 17)	− 22 (− 34, − 14)	0.018
HBPc-48 h (%) median (IQR)	− 54 (− 72, − 36)	− 15 (− 38, − 3)	< 0.001

HBP-initial, HBP level at admission. HBP-24 h, HBP level at 24 h. HBP-48 h, HBP level at 48 h. HBPc-24 h (%), changes in HBP levels between baseline and 24 h, presented as percentages. HBPc-48 h (%), changes in HBP levels between baseline and 48 h, presented as percentages.

### Comparative predictive accuracy of biomarkers

The predictive accuracy for 30-day mortality between CRP, PCT and HBP levels at admission were presented in Table 3. As compared to baseline, admission HBP demonstrated the highest predictive accuracy with an AUC of 0.79 (95% CI 0.76–0.89), followed by PCT (0.72), lactate (0.71), and CRP (0.65). Table 3 also shows that the HBPc-48 h had the highest predictive accuracy with an AUC of 0.82 (95% CI 0.75–0.89), followed by admission HBP (0.79) and HBPc-24 h (0.62).

**Table 3:** Accuracy of different biomarkers in predicting 30-day mortality.

Maximize the sum of sensitivity and specificity				
Variables	Cut-off	Sensitivity (%)	Specificity (%)	AUC (95% *CI*)
PCT (ng/dL)	1.79	0.77 (0.65, 0.86)	0.65 (0.57, 0.73)	0.72 (0.65, 0.80)
HBP (ng/mL)	201.69	0.77 (0.65, 0.86)	0.80 (0.72, 0.86)	0.79 (0.72, 0.85)
CRP	110.10	0.53 (0.40, 0.65)	0.78 (0.70, 0.85)	0.65 (0.56, 0.73)
Lactate	3.00	0.59 (0.47, 0.71)	0.75 (0.66, 0.82)	0.71 (0.63, 0.78)
HBPc-24 h (%)	− 23.26	0.63 (0.47, 0.76)	0.60 (0.52, 0.68)	0.60 (0.51, 0.70)
HBPc-48 h (%)	− 17.14	0.58 (0.43, 0.72)	0.91 (0.85, 0.95)	0.82 (0.75, 0.89)

AUC refers to the area under the ROC curves in which the larger AUC means higher discriminative capability. The cutoff was determined to maximize the sum of sensitivity and specificity. PCT, HBP, CRP and Lactate refer to their respective levels at baseline.

Supplementary Fig. 2 shows the 2 ROC curves of each predictive marker. The AUC with 95% confidence intervals, optimal cutoff points, and corresponding sensitivity and specificity are shown in Table 3. At a cutoff of − 17.14%, HBPc-48 h had a maximal specificity of 0.91 (95% CI 0.85–0.95) with a sensitivity of 0.58 (95% CI 0.43–0.72), while at a cutoff of − 57.07%, HBPc-48 h had a maximal sensitivity of 0.92 (95% CI 0.80–0.98) with a specificity of 0.45 (95% CI 0.37–0.54), which is shown in Supplementary Table 1. HBP was weakly correlated with PCT concentrations (Spearman correlation 0.21, p = 0.004, Supplemental Fig. 2).

**Figure 3:**
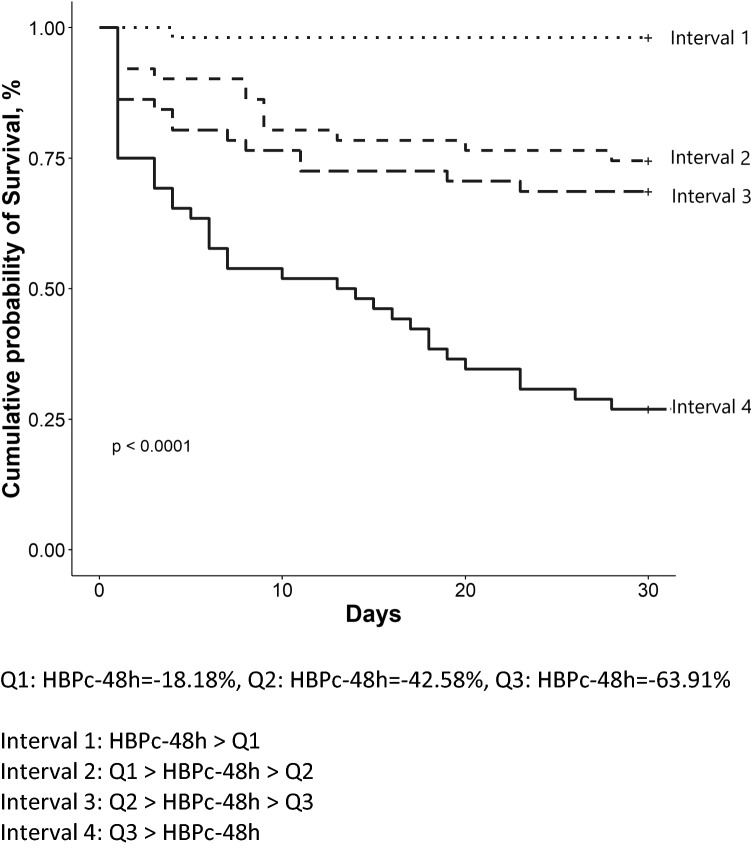
Kaplan–Meier survival curve for HBPc-48 h in four quartiles.

Our study then investigated the relationship between change in plasma HBP and survival by plotting Kaplan–Meier survival curves stratified by HBPc-48 h quartiles based on plasma HBP change between baseline and at the 48-h mark in Fig. 3, and HBPc-24 h quartiles based on plasma HBP change between baseline and at the 24-h mark in Supplemental Fig. 6. Patients with higher 48-h HBP change were associated with increased survival probability. HBPc-48 h best differentiated survivor and non-survivor groups in 30-day cumulative probability of death (p < 0.0001), while HBPc-24 h had no statistical significance in differentiating survivor and non-survivor groups (p = 0.064). For clinical use, we calculated that the patients (81 patients) with a HBPc-48 h greater than 50% had a survival rate of 91.4%, while the patients (23 patients) with a HBPc-48 h less than 4% had a mortality rate of 86.9%.

Lastly, we explored whether HBPc-48 h had incremental prognostic value in addition to commonly used clinical predictors including age, Charlson score and SOFA score. We built a Cox regression model comprising four empirical predictors. The proportional hazard assumption of Cox model was met since all predictors remained constant over time (Supplemental Table 2). We then added the quartile HBPc-48 h variable into the model. As shown in Table 4, the addition of HBPc-48 h greatly enhances the AUC of the original model from 0.85 (95% CI 0.77, 0.90) to 0.93 (95% CI 0.87, 0.95) with a DeLong test p = 0.004. The two models were all well-calibrated as shown by calibration plots. (Supplementary Fig. 4) The weight of the component variables in the two models were presented in Table 4. The hazard ratio of HBPc-48 h quartiles was 2.19 (95% Cl 1.66, 2.90, p < 0.0001) in the HBPc-48 h-enhanced model (likelihood ratio test Chi-square: 53.572, p < 0.0001). A nomogram for the HBPc-48 h enhanced model was made to calculate the predicted risk for an individual with a given set of predictors (Supplementary Fig. 5).

**Table 4:** Multivariable binary Cox regression analysis of prognosis in patients with severe sepsis or septic shock using age as a continuous variable.

Variables	HR	95% *CI*	*p*-value
Likelihood ratio test Chisq: 54.193, p-value: < 0.0001			
**Empirical model: AUC: 0.85 (0.80, 0.91)**			
Age	1.00	0.98, 1.01	0.7137
Charlson score	1.19	1.09, 1.29	< 0.0001
SOFA score	1.13	1.08, 1.17	< 0.0001
**HBPc-48 h-enhanced model: AUC: 0.93 (0.90, 0.97)**			
Age	1.00	0.99, 1.02	0.8366
HBPc-48 h quartiles	2.20	1.66, 2.91	< 0.0001
Charlson score	1.20	1.10, 1.30	< 0.0001
SOFA score	1.10	1.05, 1.14	< 0.0001

## Discussion

Among our prospective observational cohort of 206 patients with sepsis, the sepsis related 30-day mortality rate was 33.50%. HBPc-48 h was able to predict in-hospital mortality better than CRP, PCT or lactate. We found that patients with a HBPc48h greater than 50% had a 91.4% survival rate, while those with a HBPc48h less than 4% had an 86.9% mortality rate. Furthermore, HBPc-48 h showed promise for improving the accuracy of a clinical risk prediction model. These findings suggested that the change of HBP may assist in prognostication in patients with sepsis. We found that in survivors of sepsis, their plasma levels of HBP dramatically decreased at 48 h, whereas in nonsurvivors, the HBP levels decreased at a slower rate or remained elevated. We also observed that the initial plasma concentration of HBP and the HBPc-24 h poorly correlated with prognosis. The HBPc-48 h of survivors was − 53.88% (IQR − 71.83 to − 35.65), which was considerably higher than that of nonsurvivors (− 15.24%; IQR − 38.65 to − 3.13, p < 0.001). Since HBP was a strong indicator for severe sepsis which amplified vascular leakage and inflammatory responses^15^, the dynamic change of HBP may indicate initial treatment response and disease status. Further clinical trials are needed to help determine the best strategy for the use of the dynamic change of HBP information in guiding clinical treatments.

Our findings were consistent with prior studies showing that HBP was a valuable prognostic marker in patients with sepsis^17,27^. A study of 233 patients in 2009^17^ reported that plasma HBP levels of ≥ 15 ng/mL was a better indicator of severe sepsis (with or without septic shock) than PCT, IL-6, CRP, WBC and lactate. Additionally, other studies have subsequently confirmed that plasma HBP levels were significantly elevated in sepsis associated with circulatory failure^28,29^. A 2015 multicenter study of 759 patients also found that HBP could effectively predict disease progression to organ dysfunction (AUC = 0.80)^14^. Based on our study, we found that the prognostic value of HBP did not increase with time, with baseline HBP and HBPc-48 h having a higher prognostic value than HBPc-24 h. Until recently, the kinetics of HBP in patients with septic shock were not systematically studied. A recent experiment that injected HBP intravenously in rats found that a half-life below 10 min and an elimination half-life of 1–2 h. In the acute inflammation stage, HBP's rapid release and rapid elimination makes serum levels unstable^30^. Recent observations on serial changes in HBP in 12 patients with septic shock corroborated our observations. The study showed that serum HBP levels are highly variable within 72 h of admission, with baseline and peak levels having the greatest prognostic value^31^. They did not analyze the relationship between the trend in change in HBP and the outcome. Nevertheless, their findings may help explain why the baseline value has a higher prognostic value than HPB-24c, and why HBP levels at later stages are more indicative of the final outcome.

Our study has limitations. First, our study was a small single center prospective study of consecutive patients admitted to ICU with sepsis. Large prospective multicenter studies are needed to validate our results. Next, we did not collect information regarding therapeutic decision-making which includes the appropriateness of antibiotic choice for the given infection. Variance in antimicrobial resistance, source control procedures or antibiotic choice may have confounded our results. Lastly, the predictive model we built was not validated in an independent external database. However, we have proven the concept that dynamic change of HBP is potentially useful to improve the performance of a clinical prediction model. The strength of this study was that all patients included fulfilled the SEPSIS-3 criteria. This consideration reduces the potential for heterogeneous definitions of sepsis to confound our results.

## Conclusions

In our single center prospective observational trial, we found that 48-h HBP change was more accurate than CRP, PCT, lactate or initial HBP level in predicting in-hospital mortality. We observed that our patients with a 48-h HBP decrease greater than 50% had a greater than 90% chance of survival, while patients with a 48-h HBP decrease less than 4% had a nearly 90% 30-day all-cause mortality rate. The HBPc-48 h can be used to enhance the predictive accuracy of a clinical prediction model. Further studies are needed to better understand the pathophysiology of elevated HBP in septic patients and how HBP measurement may potentially inform clinical care.

## Supplementary Information

Supplementary Information.

## Data Availability

The data sets generated and/or analyzed during the current study are not publicly available due to the data confidentiality requirements of the ethics committee, but are available from the corresponding author on reasonable request and approval from the ethics committees in all institutions.

## Abbreviations

HBP: Heparin-binding protein
PCT: Procalcitonin
APACHE II score: Acute physiology and chronic health evaluation score
SOFA score: Sequential organ failure assessment score
ΔHBP: Changes in plasma heparin-binding protein
ROC curves: Receiver operating characteristic curves
HBPc-24 h: Heparin-binding protein change at 24 h
HBPc-48 h: Heparin-binding protein change at 48 h
