# Supplementary material for: Dynamic changes in heparin-binding protein as a prognostic biomarker for 30-day mortality in sepsis patients in the intensive care unit
Source: Sci Rep. 2022 Jun 24;12:10751. doi: 10.1038/s41598-022-14827-1 (PMC9232494; doi:10.1038/s41598-022-14827-1)
Supplement: Supplementary file 1 — Supplementary Information. [file 41598_2022_14827_MOESM1_ESM.pdf]

Supplementary figure 1. Time change plots of 24-hour HBP change between survivors **(A)** and nonsurvivors **(B)** and 48-hour HBP change between survivors **(C)** and nonsurvivors **(D)**

1A

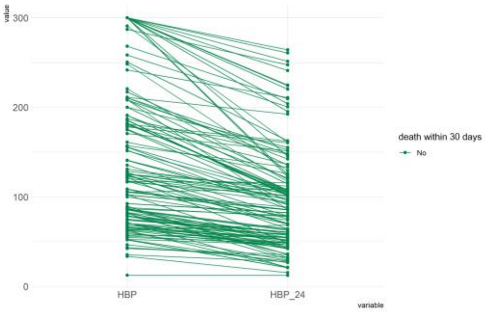

1B

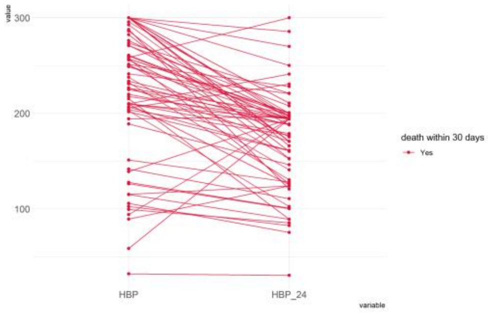

1C

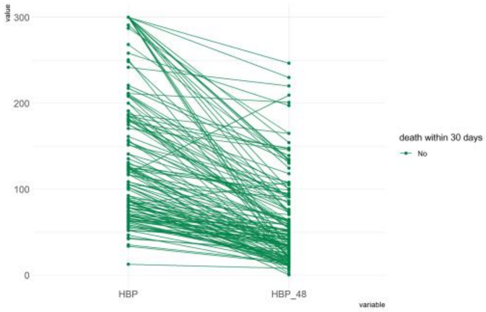

1D

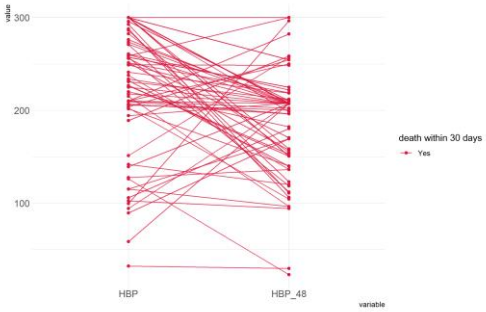

Supplementary figure 2. Receiver operating characteristic curves of PCT, CRP, Lactate, HBP, HBPc-24 and HBPc-48 **(A)** ROC curves of HBP, PCT, CRP, Lactate at baseline **(B)** ROC curves of HBP, HBPc-24, HBPc-48

(A)

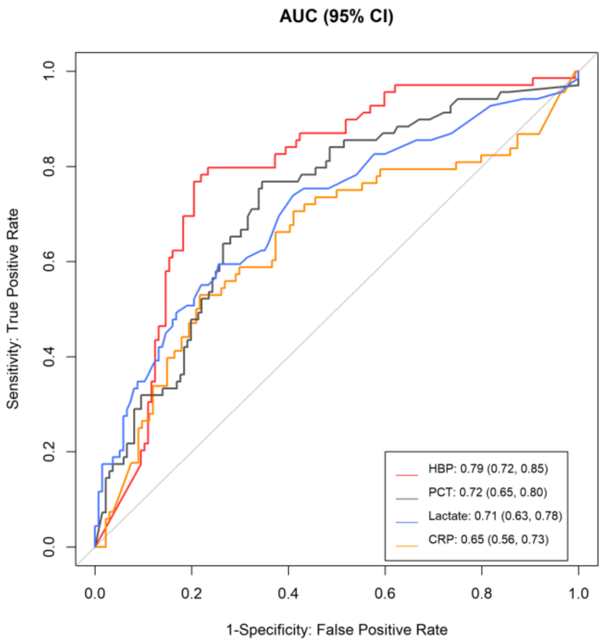

(B)

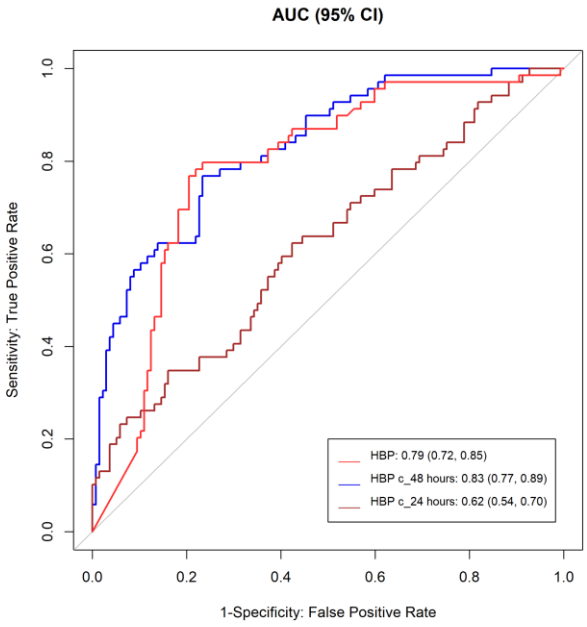

Supplementary figure 3. Correlation plot for HBP vs PCT. The Spearman rank correlation was 0.25 between HBP and PCT, P=0.0004.

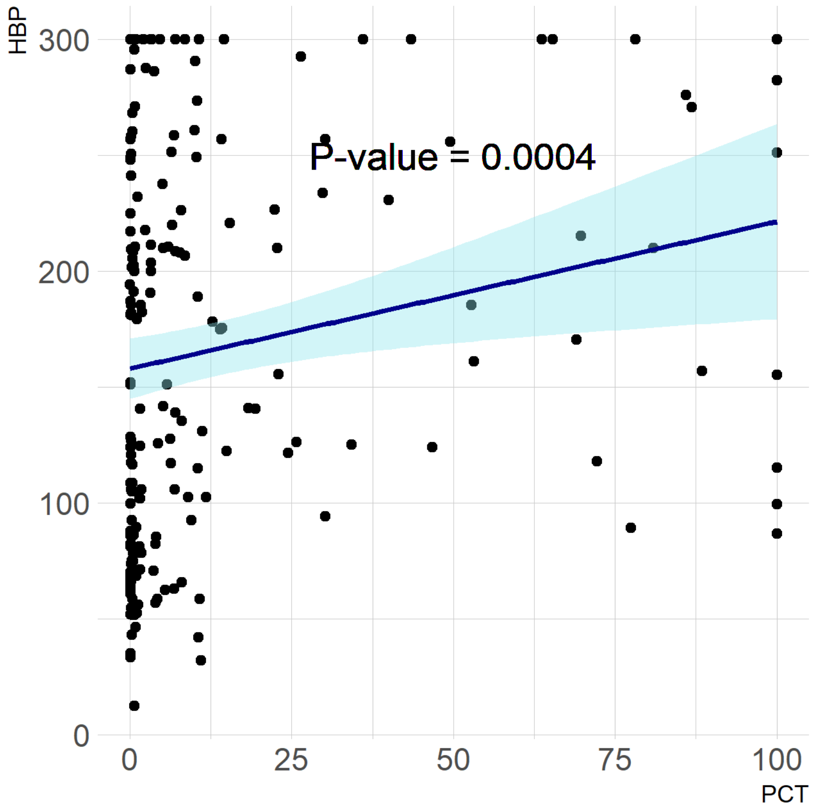

Supplementary figure 4. Calibration Plot and Brier Score
(A) Empirical model, Brier Score: 0.144, (B) HBPc-48h-enhanced model, Brier Score: 0.100

(A)

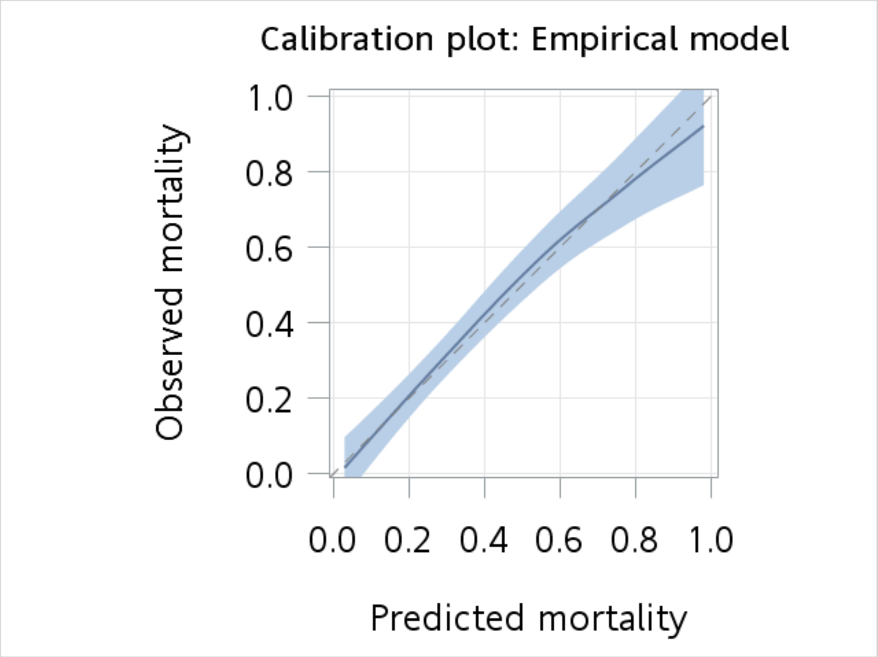

(B)

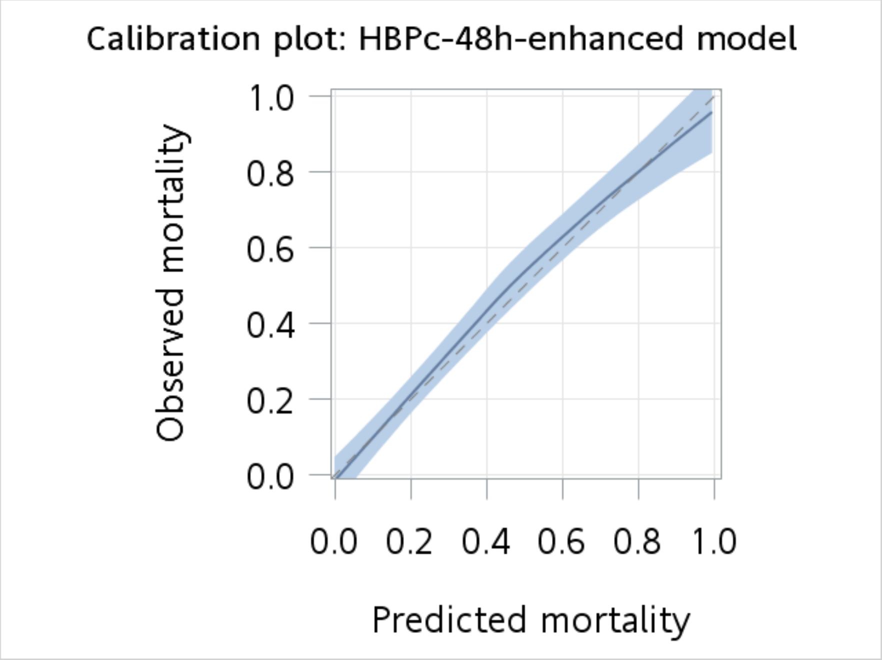

Supplementary figure 5. Nomogram of the prediction model. The relative weights of each predictor are shown in the graph which can be used to calculate an individual's risk of mortality.

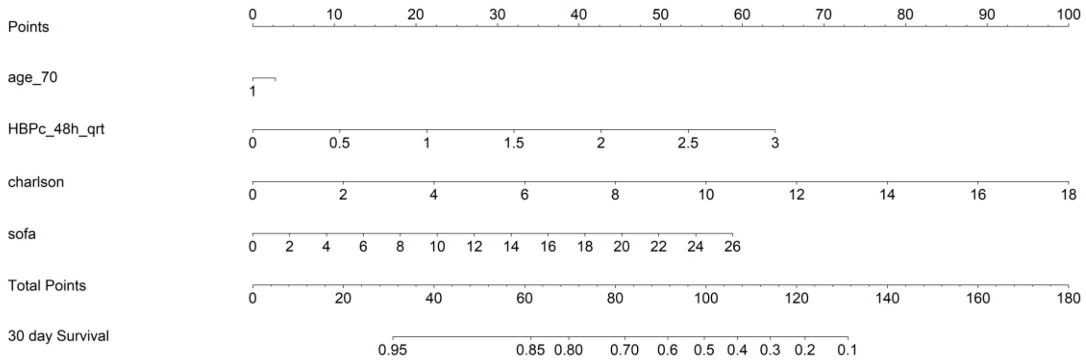

Supplementary figure 6. Kaplan-Meier survival curve for HBPc-24h in four quartiles

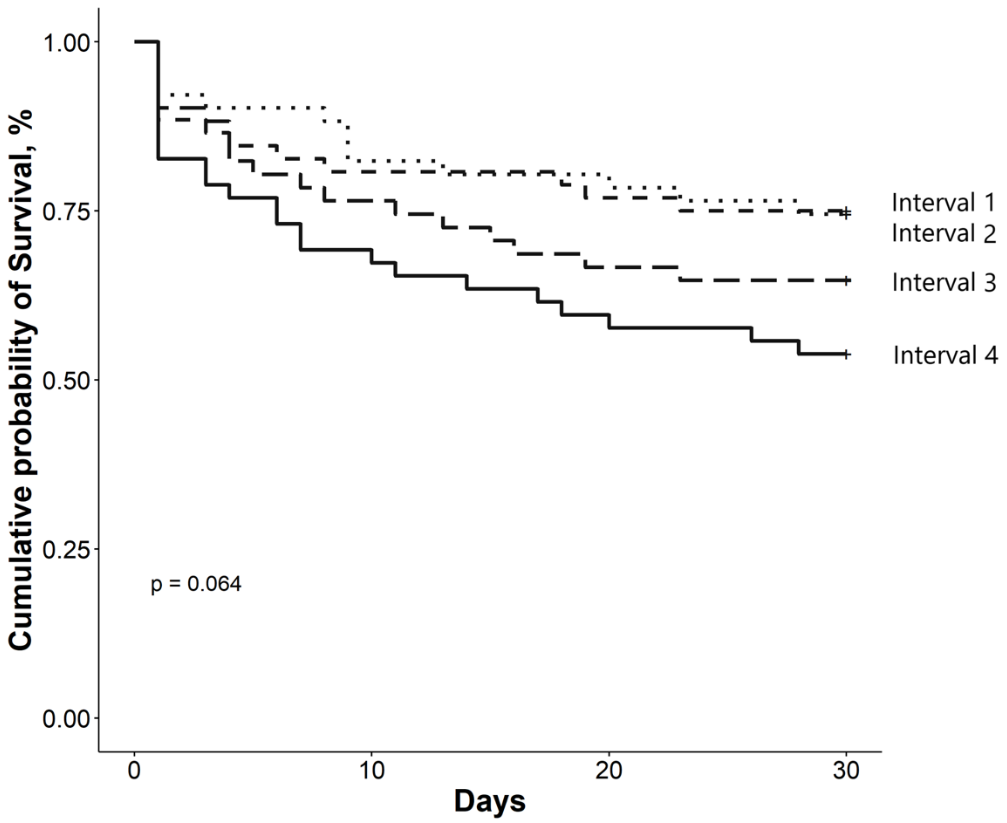

Q1: HBPc-24h=-16.26%, Q2: HBPc-24h=-25.19%, Q3: HBPc-24h=-40.48%

Interval 1: HBPc-24h > Q1
Interval 2: Q1 > HBPc-24h > Q2
Interval 3: Q2 > HBPc-24h > Q3
Interval 4: Q3 > HBPc-24h

Supplementary table 1. Accuracy of different biomarkers in predicting 30-day mortality. AUC refers to the area under the ROC curves in which the larger AUC means higher discriminative capability. The cutoff was determined to (2A) maximize sensitivity given an optimal value of specificity (0.9), and (2B) maximize specificity given an optimal value of sensitivity (0.9).

(2A) Maximize sensitivity given an optimal value of specificity (0.9)

Variables	Cut-off	Sensitivity (%)	Specificity (%)	AUC (95%CI)
PCT (ng/dL)	22.35	0.32 (0.21, 0.44)	0.90 (0.84, 0.95)	0.72 (0.65, 0.80)
HBP (ng/mL)	292.54	0.20 (0.12, 0.32)	0.91 (0.84, 0.95)	0.79 (0.72, 0.85)
CRP	163.08	0.26 (0.17, 0.39)	0.90 (0.84, 0.95)	0.65 (0.56, 0.73)
Lactate	6.00	0.35 (0.24, 0.47)	0.91 (0.85, 0.95)	0.71 (0.63, 0.78)
HBPc-24h (%)	-10.12	0.19 (0.09, 0.33)	0.94 (0.89, 0.98)	0.60 (0.51, 0.70)
HBPc-48h (%)	-17.14	0.58 (0.43, 0.72	0.91 (0.85, 0.95)	0.82 (0.75, 0.89)

(2B) Maximize specificity given an optimal value of sensitivity (0.9)

Variables	Cut-off	Sensitivity (%)	Specificity (%)	AUC (95%CI)
PCT (ng/dL)	0.23	0.91 (0.82, 0.97)	0.28 (0.21, 0.36)	0.72 (0.65, 0.80)
HBP (ng/mL)	105.80	0.91 (0.82, 0.97)	0.45 (0.36, 0.53)	0.79 (0.72, 0.85)
CRP	5.00	0.96 (0.88, 0.99)	0.04 (0.01, 0.08)	0.65 (0.56, 0.73)
Lactate	1.20	0.93 (0.84, 0.98)	0.18 (0.12, 0.26)	0.71 (0.63, 0.78)
HBPc-24h (%)	-45.98	0.92 (0.80, 0.98)	0.15 (0.10, 0.22)	0.60 (0.51, 0.70)
HBPc-48h (%)	-57.07	0.92 (0.80, 0.98)	0.45 (0.37, 0.54)	0.82 (0.75, 0.89)

Supplementary table 2. Testing the proportional hazard assumption in COX proportional hazard models

Variables	Estimates	*P*-value
Empirical model:		
Age >70 * time	0.13953	0.5259
Charlson score* time	-0.06173	0.0897
SOFA score* time	0.02769	0.1130
HBPc-48h-enhanced model:		
Age >70* time	0.07582	0.7367
HBPc-48h quartiles* time	0.07532	0.5253
Charlson score* time	-0.06199	0.0767
SOFA score* time	0.02035	0.2773

Variables	Estimates	*P*-value
Empirical model:		
Age * time	0.00596	0.2953
Charlson score* time	-0.06542	0.0793
SOFA score* time	0.02928	0.0956
HBPc-48h-enhanced model:		
Age * time	0.00609	0.4649
HBPc-48h quartiles* time	0.08696	0.3062
Charlson score* time	-0.07295	0.0466
SOFA score* time	0.02630	0.1675

Supplementary table 3. Multivariable binary Cox regression analysis of prognosis in patients with severe sepsis or septic shock using age as a categorical variable.

Variables	HR	95%*CI*	*P*-value
Likelihood ratio test<br>Chisq: 53.572, p-value:<0.0001			
Empirical model: AUC: 0.85 (0.80, 0.91)			
Age >70	0.88	0.52, 1.47	0.6143
Charlson score	1.19	1.09, 1.29	<.0001
SOFA score	1.13	1.08, 1.17	<.0001
HBPc-48h-enhanced model: AUC: 0.93 (0.90, 0.96)			
Age >70	0.94	0.56, 1.60	0.8271
HBPc-48h quartiles	2.19	1.66, 2.90	<.0001
Charlson score	1.20	1.10, 1.31	<.0001
SOFA score	1.09	1.05, 1.14	<.0001
